# *BcHTT4* Inhibits Branching of Non-Heading Chinese Cabbage at the Vegetative Stage

**DOI:** 10.3390/plants10030510

**Published:** 2021-03-09

**Authors:** Mingliang Guo, Lanlan Xu, Yan Long, Feiyi Huang, Tongkun Liu, Ying Li, Xilin Hou

**Affiliations:** 1 State Key Laboratory of Crop Genetics and Germplasm Enhancement, Ministry of Agriculture, Nanjing 210095, China; 2017204019@njau.edu.cn (M.G.); 2019104059@njau.edu.cn (L.X.); 2018104057@njau.edu.cn (Y.L.); hfy@njau.edu.cn (F.H.); liutk@njau.edu.cn (T.L.); yingli@njau.edu.cn (Y.L.); 2 College of Horticulture, Nanjing Agricultural University, Nanjing 210095, China; 3 Key Laboratory of Southern Vegetable Crop Genetic Improvement, Ministry of Agriculture, Nanjing 210095, China

**Keywords:** branching, non-heading Chinese cabbage, virus-induced gene silencing, *BcHTT4*, *FKBP13*

## Abstract

Branching is speculated to contribute to the plant architecture and crop yield. As a quantitative trait, branching is regulated by multiple genes in non-heading Chinese cabbage (NHCC). Several related candidate genes have been discovered in previous studies on the branching of NHCC, but their specific functions and regulatory mechanisms still need to be verified and explored. In this study, we found that the expression of *BcHTT4*, the ortholog to *HEAT-INDUCED TAS1 TARGET4 (HTT4)* in *Arabidopsis*, was significantly different between ‘Suzhouqing’ (common type) and ‘Maertou’ (multiple shoot branching type) in NHCC, which was consistent with the previous transcriptome sequencing results. The silencing of *BcHTT4* expression in non-heading Chinese cabbage promotes axillary bud growth at the vegetative stage. When *BcHTT4* is overexpressed in *Arabidopsis*, branching will decrease. In further study, we found that *BcHTT4* interacts with immunophilin *BcFKBP13* in vivo and in vitro through yeast two-hybrid analysis and bimolecular fluorescence complementation (BiFC) assays. Moreover, quantitative real-time PCR analysis showed that when the expression of *BcHTT4* was silenced in ‘Suzhouqing’, the expression of *BcFKBP13* also decreased significantly. Our findings reveal that *BcHTT4* is involved in the branching mechanism and interacts with immunophilin *BcFKBP13* in NHCC.

## 1. Introduction

With the increasing population in the world, the problem of food production is becoming more and more prominent and needs to be solved urgently. The appropriate number of branches and tillers plays a decisive role in the yield of most crops [1,2,3]; thus, the research on branching and tillering has received more and more attention. As a leafy vegetable, the yield of non-heading Chinese cabbage (NHCC) is mainly determined by the number of leaves. The research on the branching of NHCC not only reveals the branching mechanism from the molecular level, but also helps to improve varieties to achieve the purpose of increasing yield. However, the branching regulation of NHCC is rarely reported.

Previous studies have shown that the whole branching process was composed of axillary bud formation and subsequent growth [4,5]. In rice, sorghum, *Arabidopsis* and other plants, researchers found that tillering is affected by the interaction of developmental factors [1,6,7], hormones [8,9,10] and environmental factors [11,12]. Environmental factors, including light, temperature, water, and nutrition, form a complex network that affects the growth and development of branching. Moreover, studies have shown that the photosynthetic activity and sugar utilization rate of buds also affect the branching to a certain extent [13,14].

Previously, many genes regulating branching have been found, such as *MONOCULM 1* (*MOC1*) [4,15,16] and *DWARF27* (*D27*) [17] in rice, *LATERAL SUPPRESSOR* (*LAS*) [18,19] and *AUXIN RESISTANT 1* (*AXR1*) [20,21] in *Arabidopsis*, *TEOSINTE BRANCHED 1* (*TB1*) [1,22] in maize, and so on. As a popular and widely grown vegetable, the research on the branching of NHCC has made some progress. The NHCC, which originated from China, is divided into five varieties: var. *communis* (Tsen et Lee) Hanelt, var. *rosularis* (Tsen et Lee) Hanelt, var. *parachinensis* (L.H. Bailey) Hanelt, var. tai-tsai Hort and var. *multiceps* Hort [23]. Compared with the other four varieties, var. *multiceps* Hort has unique growth characteristics and a large number of branches are formed at the vegetative stage. However, the axillary buds of other four varieties did not begin outgrowth until the reproductive stage, which was significantly different from that of the var. *multiceps* Hort. Therefore, var. *multiceps* Hort provides an ideal material for the study of branching mechanisms of NHCC at the vegetative stage.

In previous transcriptome sequencing analysis of mixed tissues, including roots, stems, leaves, flowers, and pods of the five varieties of NHCC, we found that the expression of *BcHTT4*, the ortholog to *HEAT-INDUCED TAS1 TARGET4* (*HTT4*) in *Arabidopsis*, in var. *multiceps* Hort, was significantly different from that of the other four varieties. The expression of *BcHTT4* was down-regulated in var. *multiceps* Hort, but up-regulated in the other four varieties. This suggests that *BcHTT4* may participate in the branching regulation of NHCC to some extent, but this needs further verification. Virus-induced gene silencing (VIGS) is a kind of technology which can silence the target gene at transcription level after infecting the plant with a virus carrying the specific fragment of the target gene [24,25,26]. VIGS technology is an important tool in the study of gene function in plants. VIGS technology has been gradually used in gene function verification of NHCC, which provides a technical means for exploring the function of *BcHTT4*.

In *Arabidopsis*, *HTT4* is the target of trans-acting small interfering RNAs (ta-siRNAs), which is a unique class of small, interfering plant RNAs (siRNAs) [27,28]. The expression of *HTT4* is inhibited at the transcriptional level by complementing the sequences of target genes of siRNAs [29,30,31]. In addition, the expression of *HTT4* was up-regulated at low temperature treatment [32] in *Arabidopsis*. However, as the target of ta-siRNAs, *HTT4* encodes unknown functional proteins in *Arabidopsis* [33,34], and it has rarely been studied in other species. At present, it has been found that the expression of targets is silenced by small RNAs to regulate tillering. For example, the expression of rice SQUAMOSA promotor-binding protein-like (*OsSPL14*) gene is inhibited by microRNAs [35,36] to regulate tillering [37,38].

Here, the expression of *BcHTT4* showed opposite trends in ‘Suzhouqing’ and ‘Maertou’, which was analyzed by quantitative real-time PCR. In the functional analysis, we found that *BcHTT4* plays a negative role in branching through utilizing VIGS technology in ‘Suzhouqing’ and overexpression in *Arabidopsis*. The results of yeast two-hybrid analysis and bimolecular fluorescence complementation assays showed that *BcHTT4* interacts with *BcFKBP13* in vivo and in vitro. When the expression of *HTT4* was silenced in ‘Suzhouqing’, the expression of *FKBP13* was also down-regulated.

## 2. Results

### 2.1. BcHTT4 May Be a Negative Regulator of Branching in NHCC

**Figure 1:**
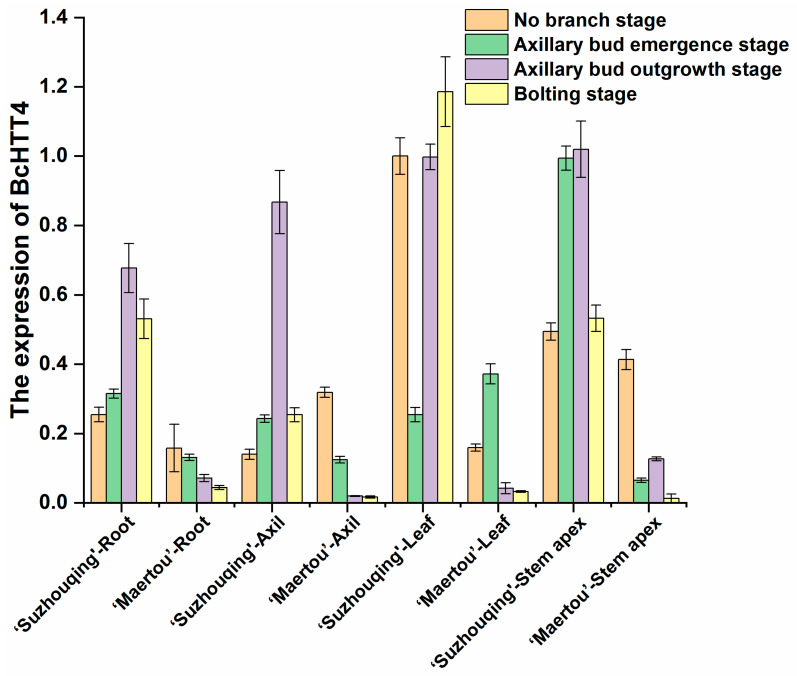
The transcription level of *BcHTT4* in the two cultivars. No branch stage (at the 40th day after germination): no obvious axillary buds appeared at the five-leaf stage in two cultivars; axillary bud emergence stage (at the 49th day after germination): apparent axillary bud just occurred in ‘Maertou’ but it is still not in ‘Suzhouqing’; axillary bud outgrowth stage (at the 80th day after germination): 70% axillary buds grown into branches in ‘Maertou’; bolting stage (at the 105th day after germination): there are flower buds generated in two cultivars [23].

According to the previous transcriptome data of mixed tissues from five varieties of NHCC, the expression of *BcHTT4* in var. *multiceps* Hort was significantly lower than that in the other four varieties. The unique branching characteristics of var. *multiceps* Hort at the vegetative stage and the difference of transcript level of *BcHTT4* between var. *multiceps* Hort and the other four varieties indicated that *BcHTT4* was related to the branching of NHCC. To further explore the expression pattern of *BcHTT4* in NHCC, we took samples at four periods of ‘Suzhouqing’ and ‘Maertou’ according to their growth characteristics and carried out quantitative real-time PCR. The quantitative real-time PCR analysis (Figure 1) showed that although *BcHTT4* was expressed in all the samples tested from ‘Suzhouqing’ and ‘Maertou’, its expression in ‘Suzhouqing’ was significantly higher than that in ‘Maertou’, which is consistent with transcriptome sequencing data. Moreover, the expression of *BcHTT4* in ‘Suzhouqing’ was significantly up-regulated with the plant growth, and the expression level reached the peak at 80 days after germination. Meanwhile, ‘Maertou’ was at the axillary bud outgrowth stage, but the expression pattern of *BcHTT4* in ‘Maertou’ was the opposite from that in ‘Suzhouqing’. The expression patterns of *BcHTT4* in ‘Suzhouqing’ and ‘Maertou’ suggest that it seems to be an inhibitor of branching in NHCC.

### 2.2. The Characteristics of BcHTT4

**Figure 2:**
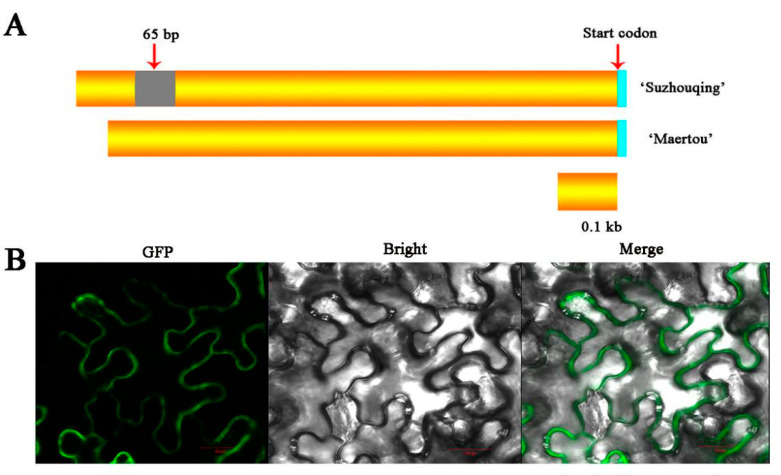
The characteristics of *BcHTT4*. (**A**) Alignment of promoter sequence of *BcHTT4* in ‘Suzhouqing’ and ‘Maertou’. (**B**) Subcellular localization of *BcHTT4*-GFP fusions in tobacco cells. The green fluorescence signal is displayed in the dark field and bright field and merged. Scale bar, 20 µm.

To verify whether the sequence of *BcHTT4* is different in ‘Suzhouqing’ and ‘Maertou’, the coding and promoter sequence of *BcHTT4* were amplified using specific primers (Table S1). Sequence alignment showed that there was no difference in the coding sequence of *BcHTT4* between the two cultivars (Figure S1A). However, the promoter sequence of *BcHTT4* was significantly different between the two cultivars. Sequence alignment showed that there was a 65 bp insertion in the *BcHTT4* promoter of the ‘Suzhouqing’ of region approximately 660 bp upstream of the start codon (Figure 2A and Figure S1B), which may have caused the expression difference of *BcHTT4* between the two cultivars. Moreover, *BcHTT4*-GFP fusions were localized in the cytoplasm and nucleus in *Arabidopsis*. We integrated GFP into the C-terminus of *BcHTT4* and transiently expressed it in tobacco cells. The result of subcellular localization showed that *BcHTT4*-GFP fusions were localized in the cell cytoplasm (Figure 2B).

### 2.3. Silencing of the BcHTT4 Expression Promotes Branching of ‘Suzhouqing’

**Figure 3:**
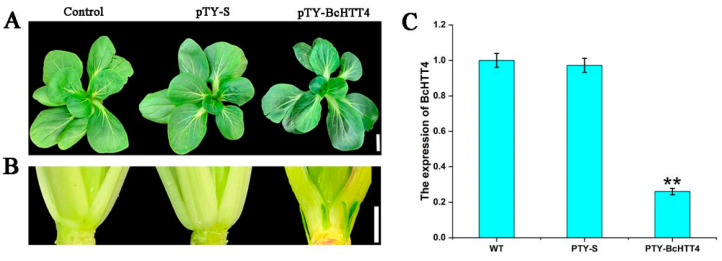
Silencing the expression of *BcHTT4* in ‘Suzhouqing’. (**A**) Phenotypes of ‘Suzhouqing’: uninfected, ‘Suzhouqing’ infected with pTY-S plasmids, and ‘Suzhouqing’ treated with pTY-*BcHTT4* plasmids. Scale bar, 2 cm. (**B**) Comparison of leaf axils among ‘Suzhouqing’: uninfected, ‘Suzhouqing’ infected with pTY-S plasmids, and ‘Suzhouqing’ infected with pTY-*BcHTT4* plasmids in plant, leaf and axil. Scale bar, 1 cm. (**C**) Transcription level of *BcHTT4* under three different treatments of ‘Suzhouqing’. Bars with double asterisks are significantly different at *p* < 0.05, according to Tukey’s single factor tests. Data are shown as the means ± SD.

The significant difference of transcription level and expression trend of *BcHTT4* between ‘Suzhouqing’ and ‘Maertou’ indicated that *BcHTT4* is a negative regulator in branching at the vegetative stage. ‘Suzhouqing’ is the representative of common cultivars; therefore, we used VIGS technology based on turnip yellow mosaic virus (TYMV) to silence the expression of *BcHTT4* in ‘Suzhouqing’, so as to explore the function of *BcHTT4* in branching at the vegetative stage. The pTY-*BcHTT4* plasmids carrying the *BcHTT4* specific fragment were used to infect ‘Suzhouqing’ plants utilizing particle bombardment at 25 days after germination. At the same time, ‘Suzhouqing’ plants were also infected by pTY-S plasmids. After 30 days of infection, the infected plants with obvious disease characteristics were obtained (Figure 3A). Through the observation of the leaf axils, we found that there were obvious axillary buds in ‘Suzhouqing’ infected by pTY-*BcHTT4* plasmids, but there was no axillary bud in ‘Suzhouqing’ which grew naturally and was infected by pTY-S plasmids (Figure 3B). Through further quantitative real-time PCR analysis, we found that the expression of *BcHTT4* decreased significantly in the plants infected with pTY-*BcHTT4* plasmids, but there was no difference in the expression of *BcHTT4* between plants which grew naturally, and plants infected by pTY-S plasmids (Figure 3C). These results showed that the silencing of *BcHTT4* promotes branching in ‘Suzhouqing’ at the vegetative stage.

### 2.4. Overexpression of BcHTT4 in Arabidopsis Resulted in Early Flowering and Branching Inhibition

**Figure 4:**
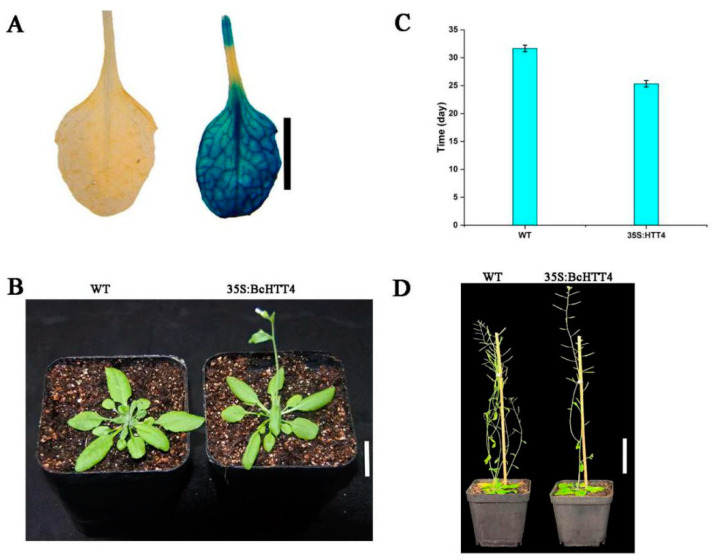
Overexpression of *BcHTT4* in *Arabidopsis*. (**A**) The β-glucuronidase (Gus) staining of *Arabidopsis*. Scale bar, 5 mm. (**B**,**C**) Comparison of bolting time between transgenic *Arabidopsis* lines and wild type *Arabidopsis*. (**B**) Scale bar, 2 cm. (**D**) Observation on branch phenotype in transgenic *Arabidopsis* lines and wild type *Arabidopsis*. Scale bar, 5 cm.

By silencing the expression of *BcHTT4* in ‘Suzhouqing’, we conclude that it plays a negative role in the branching regulation of NHCC. In order to further verify its function, we obtained *BcHTT4* overexpression *Arabidopsis* lines (Figure 4A). We found that the branches of transgenic *Arabidopsis* lines decreased sharply, which was opposite to the results of silencing *BcHTT4* in ‘Suzhouqing’ (Figure 4D). In addition, *BcHTT4*-overexpression transgenic lines showed earlier bolting and flowering, which was about six days earlier than that of wild type *Arabidopsis* (Figure 4B,C).

### 2.5. BcHTT4 Interacts with BcFKBP13

**Figure 5:**
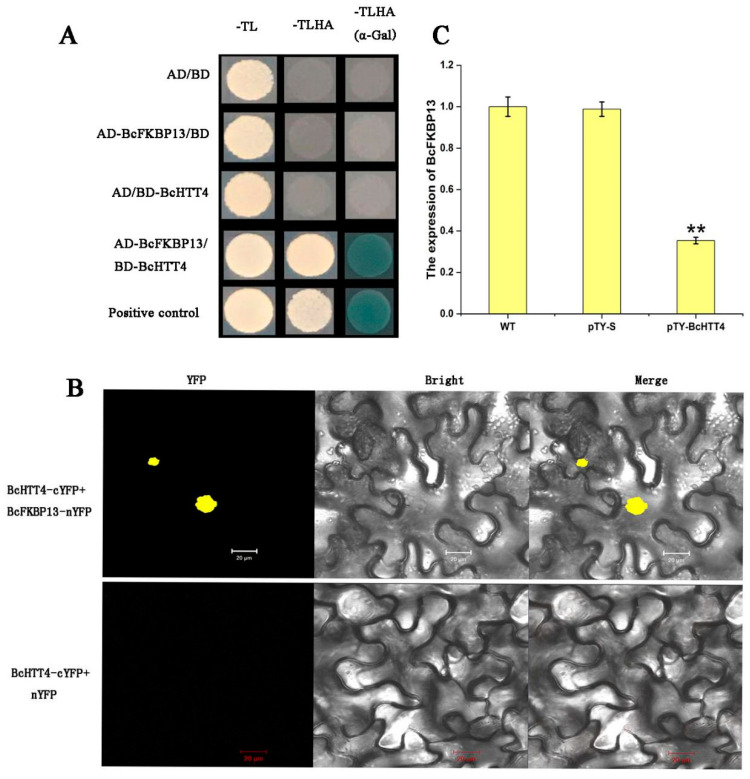
The interaction between *BcHTT4* and *BcFKBP13*. (**A**) The verification of the interaction between *BcHTT4* and *BcFKBP13* by yeast two-hybrid assay. The yeast cells were grown on the SD media: SD-TL (**left**), SD-TLHA (**middle**), SD-TLHA (added α-Gal, **right**). (**B**) Bimolecular fluorescence complementation (BiFC) analysis of the interaction between *BcHTT4* and *BcFKBP13*. Yellow fluorescence signal is displayed in a dark field and bright field, respectively, and merged. Scale bar, 20 µm. (**C**) Transcription level of *BcFKBP13* under three different treatments of ‘Suzhouqing’. Bars with double asterisks are significantly different at *p* < 0.05, according to Tukey’s single factor tests. Data are shown as the means ± SD.

In plants, the study of *HTT4* interacting protein is rare. To explore the regulatory mechanism of *BcHTT4* in NHCC, we carried out a screening library (total mRNA) by yeast two-hybrid assay. Through the screening library, we found that a gene, which was the ortholog to AtFKBP13, interacts with *BcHTT4* and was named *BcFKBP13*. The results of one-to-one verification of the yeast two-hybrid assay showed that *BcHTT4* interacts with *BcFKBP13* (Figure 5A). In addition, the yellow fluorescence in co-transformed tobacco cells expressing *BcHTT4*-cYFP and *BcFKBP13*-nYFP indicated that *BcHTT4* interacts with *BcFKBP13* in vivo through BiFC assays (Figure 5B). Taken together, these results indicate that *BcHTT4* interacts with *BcFKBP13* in vivo and in vitro. To further explore the interaction between *BcHTT4* and *BcFKBP13*, we obtained the expression level of *BcFKBP13* when the expression of *BcHTT4* was silenced in ‘Suzhouqing’ using quantitative real-time PCR. We found that when the expression of *BcHTT4* was silenced, the expression of *BcFKBP13* also decreased significantly (Figure 5C).

## 3. Discussion

Branching is an important agronomic character of plants, especially its function in yield [3,39]. Although many important genes related to branching have been found [4,18,40], there are still a lot of genes regulating branching that have not yet been discovered. Among the factors affecting branching, temperature and photosynthesis are also related to branching [13,14]. In the research of NHCC, the regulation mechanism of branching development is still in its infancy, and the gene regulation involved in branching development is rare. Our discovery of *BcHTT4* regulating branching development is a supplement to the study of branching development of NHCC.

In *Arabidopsis*, the expression of *HTT4* is regulated by temperature. The expression of *HTT4* decreased with the increase in temperature in *Arabidopsis* [30,41]. However, previous transcriptome data showed that there were significant differences in *BcHTT4* transcription levels between the branching cultivars and common cultivars. In our research, the difference of *HTT4* expression was further verified by quantitative real-time PCR in four parts, examined at four stages of ‘Suzhouqing’ and ‘Maertou’. Our study found that the transcription level of *BcHTT4* was significantly different between ‘Suzhouqing’ and ‘Maertou’, and with the occurrence and development of axillary buds, the expression of *BcHTT4* gradually increased in ‘Suzhouqing’ while the expression of *BcHTT4* showed a downward trend in ‘Maertou’, suggesting a putative negative regulator in the branching development of NHCC. Moreover, the difference of *BcHTT4* promoter between ‘Suzhouqing’ and ‘Maertou’ may be responsible for the difference of expression.

In *Arabidopsis*, the function of *HTT4* is not clear. In our study, we found that when the expression of *BcHTT4* was silenced in ‘Suzhouqing’, it promoted the branching of ‘Suzhouqing’ at the vegetative stage, and when *BcHTT4* was overexpressed in *Arabidopsis*, the number of branches was significantly reduced. These results indicated that *BcHTT4* inhibits branching. Branching has a profound effect on the yield of NHCC; improving the branching quality of NHCC is an important way to increase yield. The function of *BcHTT4* in the regulation of NHCC branching and the difference of the promoter sequence provide a theoretical basis for the yield increase in NHCC in the future. *BcHTT4* plays a negative role in the branching regulation of NHCC; thus, we can down regulate the expression of *BcHTT4* and even lose the *BcHTT4* gene function to improve the branching of NHCC. In addition, the expression of *HTT4* is closely related to temperature in *Arabidopsis* [30,41], which suggests that branching of NHCC could be affected by temperature.

Immunophilins [42,43] are found in many living cells. According to the difference of receptors, these immunophilins can be divided into two categories: cyclophilins and FKBPs (FK506 binding proteins). These receptor proteins are widely involved in many life processes [44,45,46]. In *Arabidopsis*, *FKBP13*, as an immunophilin of chloroplast thylakoid lumen [47], is involved in the transport of Rieske protein and regulates the level of Rieske protein in chloroplast thylakoid lumen through the precursor of *FKBP13* interacting with the Rieske subunit [48]. The Rieske subunit, as a part of cytochrome bf complex, plays an important role in photosynthesis. Our study showed that *BcHTT4* interacts with *BcFKBP13* by a yeast two-hybrid assay and BiFC assay. In the ‘Suzhouqing’ plants infected by pTY-*BcHTT4* plasmids, the expression of *BcFKBP13* decreased with the silencing of *BcHTT4* expression, which suggested that *BcHTT4* might promote the expression of *BcFKBP13* and affect the accumulation of Rieske protein.

In conclusion, we infer that *BcHTT4* acts as a negative regulator in the branching of NHCC at the vegetative stage, and that *BcHTT4* interacts with *BcFKBP13* in NHCC. Moreover, the precursor of *FKBP13* binds to Rieske protein and negatively regulates the accumulation of Rieske protein in *Arabidopsis*. Rieske protein belongs to the subunit of cytochrome bf complex and participates in the photosynthesis of plants. Therefore, we infer that *BcHTT4* may regulate the branching of NHCC at the vegetative stage by affecting photosynthesis; however, this needs to be further verified.

## 4. Materials and Methods

### 4.1. Plant Materials and Growth Conditions

NHCC materials included two cultivars: *B. rapa ssp. chinensis* var. *communis* Tesnet Lee, cv. ‘Suzhouqing’ (common type) and *B. rapa ssp. chinensis* var. *multiceps*, cv. ‘Maertou’ (multiple shoot branching type). The branching characters of these two materials are obviously different in the vegetative stage. Both cultivars were planted in a greenhouse under a light–dark cycle with 16 h light and 8 h dark at 25 °C.

### 4.2. Tissue Sampling of ‘Suzhouqing’ and ‘Maertou’

The roots, stem apexes, leaves and leaf axils of the two materials were sampled in four stages according to the growth and development process of the two cultivars: the 5-leaf stage in two cultivars (no branch stage); apparent axillary bud just occurred in ‘Maertou’ but it had still not in ‘Suzhouqing’ (axillary buds emergence stage); 70% axillary buds had grown into branches in ‘Maertou’ (axillary buds outgrowth stage); there are flower buds generated in two cultivars (bolting stage). RNA was then extracted and qPCR was conducted as below.

### 4.3. Quantitative Real-Time PCR

Total RNAs from sampled tissues were isolated using an RNeasy Plant Mini Kit (Qiagen) according to the user’s manual. First strand cDNA was synthesized from 5 µg of total RNA using the PrimeScript First-Strand cDNA Synthesis kit (TaKaRa, Dalian, China), according to the manufacturer’s instructions. Real-time PCR experiments were performed using a QuantiTect SYBR Green real-time-PCR kit (TaKaRa, Dalian, China) in accordance with the manufacturer’s protocols. The *BcHTT4*-specific primers QBcHTT4-F and QBcHTT4-R (Table S1) were designed using Beacon Designer 7.0 software, and β-actin (Table S1) was selected as the internal control gene. The relative expression levels of the selected transcripts were normalized to β-actin gene and calculated using the 2^−ΔΔCt^ method.

### 4.4. Acquisition of BcHTT4 Sequence

The promoter and coding sequences of *BcHTT4* in ‘Suzhouqing’ and ‘Maertou’ were amplified using specific primers (Table S1) and cloned into pEASY-Blunt Zero Cloning Vector (TranGen Biotech, China) for sequencing. Sequence alignments were performed using DNAMAN 6.0 software.

### 4.5. Subcellular Localization of BcHTT4-GFP Fusions

To construct the *BcHTT4*-GFP fusions, the coding sequence of *BcHTT4* was amplified by primers *BcHTT4*-NdeI-F and *BcHTT4*-KpnI-R (Table S1) and cloned into pRI101 vector plasmids. The *BcHTT4*-GFP plasmids were transformed into *A. tumefaciens* strain GV3101. The transformed agrobacterium strains were infected into tobacco leaves when agrobacterium cells were cultured in yeast extract broth liquid medium until the OD600 reached approximately 0.8. The infected tobacco leaves were observed under an Olympus Fluoview1000 microscope after 2–3 days of darkness [41].

### 4.6. Silencing BcHTT4 Expression by VIGS Technology

VIGS [24,25,26] technology carrying TYMV was used to study the function of *BcHTT4*. The 80 nt palindromic oligonucleotide sequence specific to *BcHTT4* named pTY-*BcHTT4* (Table S2) was fused into pTY-S plasmids to construct pTY-*BcHTT4* plasmids and were used to infect ‘Suzhouqing’ plants, avoiding affecting the expression of other ortholog genes. The amplification of pTY-*BcHTT4* plasmids of the expected size (522 nt) was used to identify positive clones by TYMV-specific primers pTYMV-F and pTYMV-R (Table S1). To infect ‘Suzhouqing’ with pTY-*BcHTT4* plasmids, 5 µg purified pTY-*BcHTT4* plasmids were diluted into 10 µL ddH_2_O, and then were used to infect ‘Suzhouqing’ plants utilizing particle bombardment. The ‘Suzhouqing’ plants that were infiltrated with pTY-S vector plasmids were regarded as controls.

### 4.7. Arabidopsis Transgenic Vector Construction and Transformation

To construction *BcHTT4* overexpression plasmids, we amplified the full-length coding sequence of *BcHTT4* with primers *BcHTT4*-clone-F and *BcHTT4*-clone-R (Table S1) and cloned it into plant overexpression vector pTCK303 using primers *BcHTT4*-BamHI-F and *BcHTT4*-SpeI-R (Table S1). Transgenic vector plasmids were transformed into *A. tumefaciens* strain GV3101, and then were cultured in yeast extract broth (YEB) liquid medium until OD600 = 1.8. The transgenic experiments were conducted through agrobacterium-mediated *Arabidopsis* transformation (floral dip) [49].

### 4.8. The GUS Staining

The X-gluc buffer was prepared and consisted of 100 mM sodium phosphate, pH 7.0, 1 mM potassium ferricyanide, 1 mM potassium ferrocyanide, 10 mM Na2-EDTA, 0.5% *v/v* Triton X-100, 20% *v/v* methanol, and 0.5 mg/mL X-gluc. Leaves from transgenic and wild type plants were incubated in X-gluc buffer at 37 °C for 12 h. Then, the leaves were immersed in 75% ethanol, incubated at room temperature to remove the chlorophyll, and photographed.

### 4.9. Yeast Two-Hybrid Assay

The coding sequences of *BcHTT4* that was amplified by primers were named *BcHTT4*-NdeI-BD-F and *BcHTT4*-EcoRI-BD-R (Table S1) and cloned into pGBKT7 vector to generate BD-*BcHTT4* fusions. The coding sequence of *BcFKBP13* was amplified by primers named *BcFKBP13*-NdeI-AD-F and *BcFKBP13*-ClaI-AD-R (Table S1) and cloned into pGADT7 vector to generate AD-*BcFKBP13* fusions. The fusion constructs, negative control plasmids AD (activation domain) and BD (binding domain), and positive control plasmids pGBKT7-53 DNA-BD and pGADT7-T were transformed into Golden Yeast (Clontech, China) cells through the lithium acetate-mediated method. The transformed yeast strains were grown on SD/-Trp-Leu (Clontech, China) medium, SD/-Leu-Trp-His-Ade (Clontech, China) medium without α-Gal, and SD/-Leu-Trp-His- Ade (Clontech, China) medium, respectively, with α-Gal at 28 °C for 2–3 days.

### 4.10. BiFC Assays in Tobacco

The coding sequence of *BcHTT4* was amplified by primers *BcHTT4*-XbaI-cYFP-F and *BcHTT4*-KpnI-cYFP-R (Table S1) and cloned into pUC-SPYCE vector to generate *BcHTT4*-cYFP plasmids. The coding sequence of *BcFKBP13* was amplified by primers *BcFKBP13*-XbaI-cYFP-F and *BcFKBP13*-KpnI-cYFP-R (Table S1) and cloned into pUC-SPYNE for generating *BcFKBP13*-nYFP. The plasmid mixtures (*BcHTT4*-cYFP and *BcFKBP13*-nYFP, *BcHTT4*-cYFP and pUC-SPYNE) were introduced into the tobacco cells. After incubation in the dark for 36 h, the yellow fluorescence signal was observed using an Olympus Fluoview1000 microscope [50].

## Data Availability

All the data are in the manuscript.

## Supplementary Materials

The following are available online at https://www.mdpi.com/2223-7747/10/3/510/s1: Table S1. The sequence of all the primers; Table S2. Sequences for construction of the VIGS vector; Figure S1. Alignment of coding region (A), and promoter sequence (B) of *BcHTT4* from ‘Suzhouqing’ and ‘Maertou’.
