# Supplementary material for: BcHTT4 Inhibits Branching of Non-Heading Chinese Cabbage at the Vegetative Stage
Source: Plants (Basel). 2021 Mar 9;10(3):510. doi: 10.3390/plants10030510 (PMC7999546; doi:10.3390/plants10030510)
Supplement: Supplementary file 1 [file plants-10-00510-s001.pdf]

plants

MDPI

Article

BcHTT4 Inhibits Branching of Non-Heading Chinese Cabbage at the Vegetative Stage

Mingliang Guo [1,2,3], Lanlan Xu [1,2,3], Yan Long [1,2,3], Feiyi Huang [1,2,3], Tongkun Liu [1,2,3], Ying Li [1,2,3] and Xilin Hou [1,2,3,*]

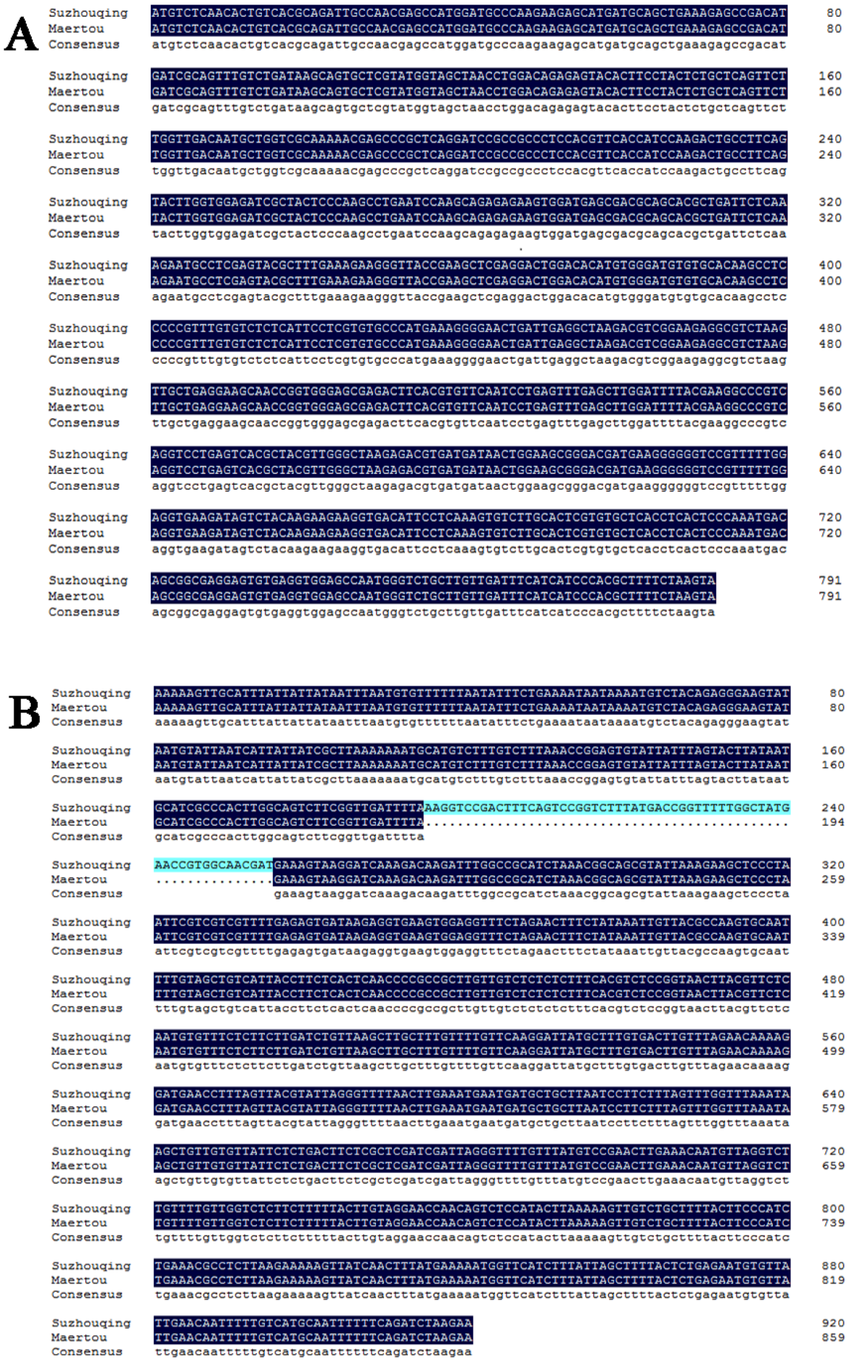

Figure 1. Alignment of coding region (A), and promoter sequence (B) of *BcTT4* from 'Suzhouqing' and 'Maertou'.

Table 1. The sequence of all the primers.

Primers for qRT-PCR Analysis	5' to 3'
β-actin-F	CTCAGTCCAAAAGAGGTATTCT
β-actin-R	GTAGAATGTGTGATGCCAGATC
Q*BcHTT4*-F	CACTTCCTACTCTGCTCAG
Q*BcHTT4*-R	GTCGCTCATCCACTTCTC
Q*Bc*FKBP13-F	GGACCAGGGAATCATTGGCT
Q*Bc*FKBP13-R	ATGAGCCTCCTTTGCAACCT
**Primers for gene cloning**	
BcHTT4-clone-F	ATGTCTCAACACTGTCACGCAGA
BcHTT4-clone-F	TTACTTAGAAAAGCGTGGGATGATG
**Primers for overexpression vector construction**	
BcHTT4-BamHI-F	GGATCCATGTCTCAACACTGTCACGCAGA
BcHTT4-SpeI-R	ACTAGTCTTAGAAAAGCGTGGGATGATG
**Primers for yeast two-hybrid**	
BcHTT4-NdeI-BD-F	CATATGATGTCTCAACACTGTCACGCAGA
BcHTT4-EcoRI-BD-R	GAATTCCTTAGAAAAGCGTGGGATGATG
*Bc*FKBP13-NdeI-AD-F	CATATGATGAGCTCCTTAGGATTTTCTGTTG
*Bc*FKBP13-ClaI-AD-R	ATCGATCAGCTTTCCCTATGAACTCAATG
**Primers for yeast BIFC assays**	
BcHTT4-XbaI-cYFP-F	TCTAGAATGTCTCAACACTGTCACGCAGA
BcHTT4-KpnI-cYFP-R	GGTACCCTTAGAAAAGCGTGGGATGATG
*Bc*FKBP13-XbaI-cYFP-F	TCTAGAATGAGCTCCTTAGGATTTTCTGTTG
*Bc*FKBP13-KpnI-cYFP-R	GGTACCAGCTTTCCCTATGAACTCAATG
**Primers for VIGS test**	
pTYMV-F	TCCACCCTCACCACCTTC
pTYMV-R	GGGACAGACCTCGCTAACT
**Primers for BcHTT4-GFP fusions**	
BcHTT4-NdeI-F	CATATGATGTCTCAACACTGTCACGCAGA
BcHTT4-KpnI-R	GGTACCCTTAGAAAAGCGTGGGATGATG

Table 2. Sequences for construction of the VIGS vector.

Sequences for Construction of VIGS Vector (5' to 3')
BcHTT4-pTY:
TTGACAATGCTGGTCGCAAAAACGAGCCCGCTCAGGATCCGGATCCTGAGCGGGCTCGTTTTTGCGACCAGCATTGTCAA
